# Antimicrobial susceptibility among gram-negative isolates collected in the USA between 2005 and 2011 as part of the Tigecycline Evaluation and Surveillance Trial (T.E.S.T.)

**DOI:** 10.1186/1476-0711-12-24

**Published:** 2013-09-05

**Authors:** Gerald A Denys, Steven M Callister, Michael J Dowzicky

**Affiliations:** 1 Indiana University Health Pathology Laboratory, Indianapolis 46202, IN, USA; 2 Microbiology Research and Molecular Diagnostics Laboratory, Gundersen Health System, La Crosse, Wisconsin 54601, USA; 3 Pfizer Inc, 500 Arcola Road, E-Dock, Collegeville 19426, PA, USA

**Keywords:** Surveillance, Tigecycline, Resistance, USA, Census regions

## Abstract

**Background:**

The Tigecycline Evaluation and Surveillance Trial (T.E.S.T.) was designed to monitor *in vitro* antimicrobial susceptibility to tigecycline and comparator agents. We present susceptibility data on Gram-negative organisms collected between 2005 and 2011 from nine United States census regions.

**Methods:**

T.E.S.T. was conducted using standardized CLSI methodologies or FDA-approved breakpoints.

**Results:**

Tigecycline was highly active (MIC_90_ ≤ 2 mg/L) against Enterobacteriaceae irrespective of species or region of collection (N = 25011). The isolates were also highly susceptible to the carbapenems when all regional data are combined, except for ESBL-producing *Klebsiella pneumoniae* (MIC_90_ 16 mg/L) and *Acinetobacter baumannii* (MIC_90_ ≥ 32 mg/L). In addition, 883 (30%) of 2900 *A. baumannii* isolates were classified as multidrug-resistant (MDR): these MDR organisms were most susceptible to tigecycline (MIC_90_ 2 mg/L) and minocycline (MIC_90_ 8 mg/L) when all regional data are considered together. Susceptibility patterns also varied widely among the regions

**Conclusions:**

The findings highlight the importance of monitoring antimicrobial susceptibility patterns and implementing effective methods to curb increased resistance and also confirm that additional studies to determine the efficacy of tigecycline *in vivo*, especially for treating infections with MDR organisms, are warranted.

## Background

Infection with *Acinetobacter* spp. and some members of the Enterobacteriaceae present clinicians with considerable challenge, especially since resistance to carbapenems is becoming increasingly prevalent [1,2]. Such infections result in increased mortality and morbidity, and the increased hospitalization costs continue to put enormous strain on the healthcare system [3-5]. Against this background, surveillance studies designed to monitor antimicrobial resistance of Gram-negative bacteria collected from regions throughout the USA are essential.

Tigecycline is a novel glycylcycline antimicrobial that overcomes several common mechanisms used by bacteria to develop resistance [6]. Confirmation of the effectiveness of tigecycline against a broad spectrum of microorganisms resulted in licensing by the US Food and Drug Administration (FDA) for the treatment of complicated skin and skin structure infections (cSSSIs), intra-abdominal infections (cIAIs), and community-acquired bacterial pneumonia (CAP) [7].

The Tigecycline Evaluation and Surveillance Trial (T.E.S.T.) is a multi-center study designed to monitor the *in vitro* activity of tigecycline and a wide range of other antimicrobial agents against clinically-important Gram-positive and Gram-negative pathogens collected globally. This report focuses on data obtained from Gram-negative organisms collected in the USA. In a previous report, Halstead et al. [8] confirmed significant *in vitro* activity of tigecycline against *A. calcoaceticus-baumannii* complex, *Enterobacter* spp., *Escherichia coli*, and *Klebsiella pneumoniae* isolates and highlighted the ability of local susceptibility patterns to more effectively guide empiric antimicrobial therapy. This study reports the *in vitro* activity of tigecycline against *A. baumannii* and other Enterobacteriaceae isolates. In addition, the susceptibility patterns among nine distinct regions within the USA are presented and emerging trends in resistance are evaluated by comparing the results to previous findings [8].

## Methods

### Isolate collection

Gram-negative isolates were collected between 2005 and 2011 from 173 centers that were divided into nine census regions: East North Central, 31; East South Central, 11; Middle Atlantic, 44; Mountain, 7; New England, 7; Pacific, 11; South Atlantic, 33; West North Central, 16; West South Central, 13. The states contained in each census region are shown in Table 1.

**Table 1:** **Numbers of isolates contributed by census region**^**a **^**in T.E.S.T.**

**Pathogen**	**Pacific**	**Mountain**	**West North Central**	**East North Central**	**Middle Atlantic**	**New England**	**South Atlantic**	**East South Central**	**West South Central**	**Total**
*A. baumannii*	103	72	332	694	721	77	562	162	177	2900
*E. coli*	240	235	813	1477	1892	226	1248	391	398	6920
*K. pneumoniae*	181	164	590	1174	1518	170	1096	331	311	5535
*K. oxytoca*	57	50	176	301	279	45	145	49	68	1170
*S. marcescens*	87	61	302	544	609	67	475	140	136	2421
*Enterobacter* spp.	227	192	770	1351	1500	198	1118	339	370	6065
**Total**	895	774	2983	5541	6519	783	4644	1412	1460	25011

^a^ Pacific = California, Hawaii, Oregon, and Washington; Mountain = Arizona, Colorado, New Mexico, Montana, and Utah; West North Central = Iowa, Kansas, Minnesota, Missouri, Nebraska, North Dakota, and South Dakota; East North Central = Indiana, Illinois, Michigan, Ohio, and Wisconsin; Middle Atlantic = New Jersey, New York, and Pennsylvania; New England = Connecticut, Maine, Massachusetts, New Hampshire, and Vermont; South Atlantic = Delaware, District of Columbia, Florida, Georgia, Maryland, North Carolina, South Carolina, Virginia, and West Virginia; East South Central = Alabama, Kentucky, Mississippi, and Tennessee; West South Central = Arkansas, Louisiana, Oklahoma, and Texas.

The centers submitted clinically-significant (determined by local criteria) Gram-negative isolates that were collected consecutively. The organisms included *Acinetobacter* spp., *E. coli*, *Enterobacter* spp., *Serratia* spp., and *Klebsiella* spp. A single isolate was collected from each patient and inclusion was independent of medical history, previous antimicrobial use, sex or age of the patient.

Organisms were identified using routine methodologies practiced regularly at each institution. Prior to eligibility for participation, the medical centers were evaluated by the central laboratory (IHMA: Laboratories International for Microbiology Studies, a division of International Health Management Associates, Inc. [IHMA, Schaumburg, IL, USA]) for adherence to national guidelines. In addition, IHMA confirmed the identification of organisms or antimicrobial susceptibility patterns using RapidOne and/or RapidNF identification systems (Remel, Lenexa, KS) when the forwarded results were uncharacteristic.

### Susceptibility testing

Minimum inhibitory concentrations (MICs) were determined using broth microdilution methodology, Sensititre® plates (TREK Diagnostic Systems, West Sussex, England) or MicroScan® panels (Siemens, Sacramento, CA, USA). Susceptibility to amikacin, amoxicillin-clavulanate, ampicillin, carbapenems (imipenem/meropenem), cefepime, ceftazidime, ceftriaxone, levofloxacin, minocycline, and piperacillin-tazobactam were interpreted according to the guidelines published by the Clinical and Laboratory Standards Institute [9-11]. In addition, susceptibility to imipenem was evaluated on isolates collected through 2006; meropenem was substituted thereafter due to imipenem stability issues. MIC values against tigecycline were evaluated using FDA-approved breakpoints provided in the package insert [7]. Quality control strains were *E. coli* ATCC 25922 and *P. aeruginosa* ATCC 27853. Results are presented as MIC_90_.

### Extended-spectrum β -lactamase (ESBL) testing

Extended spectrum β-lactamase (ESBL) production by *E. coli* or *Klebsiella* spp. was also confirmed using accepted methodology [10]. Briefly, discs that contained cefotaxime (30 μg), cefotaxime/clavulanic acid (30/10 μg), ceftazidime (30 μg), or ceftazidime/clavulanic acid (30/10 μg) (Oxoid, Inc., Ogdensburg, NY, USA) were placed onto Mueller-Hinton agar (Remel, Inc., Lenexa, KS) plates after they were overlaid with the isolate. Organisms where the combination of cefotaxime and ceftazidine discs yielded a zone of inhibition larger by >5 mm than the zone of inhibition for cefotaxime or ceftazidime were considered ESBL-producers. *K. pneumoniae* ATCC 700603 (ESBL-positive) and *E. coli* ATCC 25922 (ESBL-negative) were included for quality control.

### Statistical analyses

The Fisher’s Exact test (SAS, Version 8.2) was used to assess the relationships between the susceptibility/non-susceptibility results presented in the current report compared with previous T.E.S.T. study data. Comparisons that yielded p values ≤ 0.01 were considered significant.

## Results

### *Acinetobacter baumannii*

A total of 2900 isolates of *A. baumannii* were evaluated (Table 2). Regardless of the region, the MIC_90_ for tigecycline was ≤ 2 mg/L (MIC_50_ 0.5 mg/L), but formal conclusion regarding susceptibility was not possible because breakpoint values have not been established. The isolates were also highly susceptible to minocycline (84.1%). Carbapenem susceptibility ranged from 50% in East North Central to 80% in West South Central. In addition, some susceptibility patterns varied significantly among census regions. For example, there was dramatic regional variation in the numbers of isolates susceptible to amikacin in the East North Central (58.8% susceptible) region compared to the numbers obtained from the New England (100% susceptible) region.

**Table 2:** **Antimicrobial susceptibility for *Acinetobacter baumannii *and multidrug-resistant (MDR) *A. baumannii***

		**Pacific**	**Mountain**	**West North Central**	**East North Central**	**Middle Atlantic**	**New England**	**South Atlantic**	**East South Central**	**West South Central**	**USA**
***Acinetobacter baumannii***		N = 103	N = 72	N = 332	N = 694	N = 721	N = 77	N = 562	N = 162	N = 177	N = 2900
**Amikacin**	**MIC**_**50**_	4	4	4	8	4	4	4	4	4	4
	**MIC**_**90**_	≥128	≥128	≥128	≥128	64	8	64	≥128	≥128	≥128
	**%S**	71.8	69.4	80.4	58.8	78.1	100	85.1	69.8	71.2	74.3
**Carbapenems**	**MIC**_**50**_	1	1	1	4	1	1	1	2	1	1
	**MIC**_**90**_	16	≥32	≥32	≥32	≥32	≥32	≥32	≥32	≥32	≥32
	**%S**	76.7	69.4	74.7	50.3	70.3	74.0	74.0	66.7	80.2	67.4
**Cefepime**	**MIC**_**50**_	16	8	8	16	16	16	8	32	8	16
	**MIC**_**90**_	≥64	≥64	≥64	≥64	≥64	≥64	≥64	≥64	≥64	≥64
	**%S**	46.6	55.6	55.7	38.2	42.7	48.1	52.8	40.1	55.9	46.3
**Ceftazidime**	**MIC**_**50**_	16	≤8	≤8	≥64	32	16	16	≥64	≤8	32
	**MIC**_**90**_	≥64	≥64	≥64	≥64	≥64	≥64	≥64	≥64	≥64	≥64
	**%S**	48.5	55.6	52.4	35.6	43.8	45.5	49.6	37.0	57.6	44.9
**Ceftriaxone**	**MIC**_**50**_	32	16	16	64	32	32	32	≥128	16	32
	**MIC**_**90**_	≥128	≥128	≥128	≥128	≥128	≥128	≥128	≥128	≥128	≥128
	**%S**	30.1	30.6	35.8	19.3	25.8	22.1	27.0	20.4	31.6	25.9
**Levofloxacin**	**MIC**_**50**_	0.5	0.25	0.25	8	8	4	2	8	1	4
	**MIC**_**90**_	≥16	≥16	≥16	≥16	≥16	≥16	≥16	≥16	≥16	≥16
	**%S**	55.3	62.5	56.9	33.1	40.5	46.8	51.1	35.8	53.7	44.4
**Minocycline**	**MIC**_**50**_	≤0.5	≤0.5	≤0.5	1	≤0.5	1	1	2	≤0.5	≤0.5
	**MIC**_**90**_	4	8	8	8	8	4	8	8	8	8
	**%S**	91.3	80.6	87.3	80.5	88.2	97.4	81.5	68.5	88.7	84.1
**Pip-taz**	**MIC**_**50**_	16	8	4	128	16	8	16	64	16	16
	**MIC**_**90**_	≥256	≥256	≥256	≥256	≥256	≥256	≥256	≥256	≥256	≥256
	**%S**	53.4	56.9	59.9	36.5	53.8	61.0	54.4	43.8	59.3	50.5
**Tigecycline**	**MIC**_**50**_	0.5	0.5	0.5	0.5	0.5	0.5	0.5	0.5	0.5	0.5
	**MIC**_**90**_	1	1	1	2	2	1	2	1	2	2
	**%S**	--	--	--	--	--	--	--	--	--	--
**MDR*Acinetobacter baumannii***		N = 28	N = 23	N = 72	N = 326	N = 206	N = 13	N = 124	N = 50	N = 41	N = 883
**Amikacin**	**MIC**_**50**_	≥128	≥128	64	≥128	32	4	32	≥128	64	64
	**MIC**_**90**_	≥128	≥128	≥128	≥128	≥128	8	≥128	≥128	≥128	≥128
	**%S**	10.7	8.7	36.1	16.0	42.2	100	40.3	6.0	12.2	27.3
**Carbapenems**	**MIC**_**50**_	16	≥32	≥32	≥32	≥32	≥32	≥32	≥32	≥32	≥32
	**MIC**_**90**_	≥32	≥32	≥32	≥32	≥32	≥32	≥32	≥32	≥32	≥32
	**%S**	21.4	8.7	15.3	4.6	12.1	0.0	1.6	10.0	26.8	8.7
**Cefepime**	**MIC**_**50**_	≥64	≥64	≥64	≥64	≥64	32	32	≥64	32	≥64
	**MIC**_**90**_	≥64	≥64	≥64	≥64	≥64	≥64	≥64	≥64	≥64	≥64
	**%S**	0.0	4.3	4.2	10.4	2.4	0.0	5.6	2.0	2.4	5.9
**Ceftazidime**	**MIC**_**50**_	≥64	≥64	≥64	≥64	≥64	≥64	≥64	≥64	≥64	≥64
	**MIC**_**90**_	≥64	≥64	≥64	≥64	≥64	≥64	≥64	≥64	≥64	≥64
	**%S**	3.6	4.3	1.4	9.2	3.4	0.0	4.0	2.0	0.0	5.2
**Ceftriaxone**	**MIC**_**50**_	≥128	≥128	≥128	≥128	≥128	≥128	≥128	≥128	≥128	≥128
	**MIC**_**90**_	≥128	≥128	≥128	≥128	≥128	≥128	≥128	≥128	≥128	≥128
	**%S**	3.6	0.0	0.0	0.9	0.5	0.0	0.8	0.0	0.0	0.7
**Levofloxacin**	**MIC**_**50**_	≥16	8	8	≥16	≥16	≥16	≥16	≥16	≥16	≥16
	**MIC**_**90**_	≥16	≥16	≥16	≥16	≥16	≥16	≥16	≥16	≥16	≥16
	**%S**	0.0	4.3	2.8	0.9	2.4	0.0	0.0	0.0	0.0	1.2
**Minocycline**	**MIC**_**50**_	1	4	4	2	2	2	4	4	4	2
	**MIC**_**90**_	8	8	16	8	16	4	8	8	8	8
	**%S**	82.1	56.5	69.4	77.9	72.3	92.3	65.3	54.0	68.3	72.1
**Pip-taz**	**MIC**_**50**_	≥256	≥256	≥256	≥256	128	128	≥256	≥256	≥256	≥256
	**MIC**_**90**_	≥256	≥256	≥256	≥256	≥256	≥256	≥256	≥256	≥256	≥256
	**%S**	3.6	4.3	8.3	3.1	10.7	15.4	3.2	2.0	2.4	5.4
**Tigecycline**	**MIC**_**50**_	1	1	1	1	1	1	1	1	1	1
	**MIC**_**90**_	2	1	2	2	2	4	2	2	2	2
	**%S**	--	--	--	--	--	--	--	--	--	--

Amoxicillin-clavulanate and ampicillin are not presented in this table as they are inactive against *A. baumannii.*

% S, % susceptible, *pip-taz* piperacillin-tazobactam, carbapenems = imipenem/meropenem.

-- No CLSI approved interpretive criteria availableData not presented when N < 10 isolates.

A total of 883 (30.4%) *A. baumannii* isolates were multidrug resistant (MDR, resistant to three or more classes of antimicrobial agent [β-lactams, aminoglycosides, carbapenems or fluoroquinolones]) and the frequencies ranged from 16.9% (13/77) in the New England region to 47.0% (326/694) in the East North Central region. Furthermore, MDR isolates were susceptible to minocycline (MIC_50_ 2 mg/L, MIC_90_ 8 mg/L) in some instances, but minocycline-nonsusceptible isolates were commonly recovered from the East South Central (54% susceptibility) and Mountain regions (56.5%) (Table 2). In contrast, MDR *A. baumannii* appeared universally susceptible to tigecycline (MIC_50_ 1 mg/L, MIC_90_ 2 mg/L).

### *Escherichia coli*

Non-ESBL-producing *E. coli* isolates (n = 6643) were highly susceptible to amikacin, carbapenems, cefepime, ceftriaxone, and tigecycline (Table 3). In contrast, the organisms were significantly less susceptible to ampicillin, and the frequency of resistant organisms varied widely by region (e.g. susceptibility rates of 41.4% in East South Central region, 54.1% in the West North Central region). *E. coli* isolates that produced ESBL were also relatively uncommon (277 [4.0%] of 6920 isolates), but the highest frequency was detected in isolates from the Mountain region (11.1%, 26/235). In addition, ESBL producers were highly susceptible to amikacin (MIC_50_ 4 mg/L, MIC_90_ 16 mg/L, 94.9% susceptible), carbapenems (MIC_50_ ≤ 0.06 mg/L, MIC_90_ 0.25 mg/L, 98.2% susceptible), and tigecycline (MIC_50_ 0.25 mg/L, MIC_90_ 0.5 mg/L, 100% susceptible) (Table 4).

**Table 3:** Antimicrobial susceptibility for Enterobacteriaceae

		**Pacific**	**Mountain**	**West North Central**	**East North Central**	**Middle Atlantic**	**New England**	**South Atlantic**	**East South Central**	**West South Central**	**USA**
***E. coli*(ESBL negative)**		N = 230	N = 209	N = 801	N = 1411	N = 1811	N = 220	N = 1192	N = 384	N = 385	N = 6643
**Amikacin**	**MIC**_**50**_	2	2	2	2	2	2	2	2	2	2
	**MIC**_**90**_	4	8	4	4	4	4	4	8	4	4
	**%S**	99.6	99.0	100	99.5	99.3	99.5	99.0	98.2	99.7	99.4
**Amoxi-clav**	**MIC**_**50**_	4	8	4	8	8	4	4	8	8	8
	**MIC**_**90**_	16	32	16	16	16	32	32	32	16	16
	**%S**	77.0	71.8	80.8	75.7	76.1	76.4	75.4	70.1	76.4	76.0
**Ampicillin**	**MIC**_**50**_	4	≥64	4	32	≥64	8	≥64	≥64	≥64	32
	**MIC**_**90**_	≥64	≥64	≥64	≥64	≥64	≥64	≥64	≥64	≥64	≥64
	**%S**	52.6	44.0	54.1	47.4	46.3	51.4	45.4	41.4	43.4	47.2
**Carbapenems**	**MIC**_**50**_	≤0.06	≤0.06	≤0.06	≤0.06	≤0.06	≤0.06	≤0.06	≤0.06	≤0.06	≤0.06
	**MIC**_**90**_	0.25	0.25	0.25	0.12	0.25	0.25	0.25	0.25	0.25	0.25
	**%S**	100	99.0	99.6	99.7	99.6	99.1	99.1	97.7	99.7	99.4
**Cefepime**	**MIC**_**50**_	≤0.5	≤0.5	≤0.5	≤0.5	≤0.5	≤0.5	≤0.5	≤0.5	≤0.5	≤0.5
	**MIC**_**90**_	≤0.5	≤0.5	≤0.5	≤0.5	≤0.5	≤0.5	≤0.5	≤0.5	≤0.5	≤0.5
	**%S**	99.1	99.0	99.6	99.1	98.8	99.1	98.5	98.2	99.5	99.0
**Ceftriaxone**	**MIC**_**50**_	≤0.06	≤0.06	≤0.06	≤0.06	≤0.06	≤0.06	≤0.06	≤0.06	≤0.06	≤0.06
	**MIC**_**90**_	0.12	1	0.12	0.25	0.25	0.25	0.25	0.5	0.12	0.25
	**%S**	97.0	90.4	96.9	96.7	93.9	93.2	94.3	91.1	96.4	94.9
**Levofloxacin**	**MIC**_**50**_	0.03	0.03	0.03	0.03	0.03	0.03	0.03	0.03	0.03	0.03
	**MIC**_**90**_	≥16	≥16	≥16	≥16	≥16	≥16	≥16	≥16	≥16	≥16
	**%S**	77.8	76.1	79.7	74.7	72.5	78.6	70.6	72.1	74.0	74.0
**Minocycline**	**MIC**_**50**_	1	1	1	1	1	1	1	1	1	1
	**MIC**_**90**_	8	16	8	8	8	8	8	8	8	8
	**%S**	86.1	80.4	89.6	84.5	83.4	83.6	85.2	85.9	86.0	85.0
**Pip-taz**	**MIC**_**50**_	1	1	1	1	1	1	1	1	1	1
	**MIC**_**90**_	4	4	4	4	4	4	4	4	4	4
	**%S**	95.2	94.3	97.5	96.7	97.1	96.8	96.1	96.9	97.7	96.7
**Tigecycline**	**MIC**_**50**_	0.12	0.12	0.12	0.12	0.12	0.12	0.12	0.12	0.12	0.12
	**MIC**_**90**_	0.25	0.25	0.25	0.5	0.5	0.25	0.25	0.25	1	0.5
	**%S**	100	100	99.9	100	99.9	100	100	100	100	100
***K. pneumoniae*(ESBL negative)**		N = 159	N = 151	N = 575	N = 1061	N = 1250	N = 150	N = 991	N = 320	N = 294	N = 4951
**Amikacin**	**MIC**_**50**_	2	1	1	1	2	1	2	1	1	1
	**MIC**_**90**_	2	2	2	2	8	2	2	2	2	2
	**%S**	100	98.7	99.5	99.8	97.2	99.3	98.6	99.1	99.0	98.7
**Amoxi-clav**	**MIC**_**50**_	2	2	2	2	2	2	2	2	2	2
	**MIC**_**90**_	8	16	8	8	32	8	16	16	8	16
	**%S**	95.0	88.7	93.6	90.8	82.2	90.0	87.4	89.1	91.2	88.2
**Ampicillin**	**MIC**_**50**_	32	≥64	32	≥64	≥64	≥64	≥64	≥64	32	≥64
	**MIC**_**90**_	≥64	≥64	≥64	≥64	≥64	≥64	≥64	≥64	≥64	≥64
	**%S**	2.5	0.7	1.9	1.2	2.6	4.0	1.7	1.3	1.7	1.9
**Carbapenems**	**MIC**_**50**_	≤0.06	≤0.06	≤0.06	≤0.06	≤0.06	≤0.06	≤0.06	≤0.06	≤0.06	≤0.06
	**MIC**_**90**_	0.12	0.5	0.25	0.25	0.5	0.12	0.25	0.12	0.5	0.25
	**%S**	100	97.4	99.5	98.8	92.5	99.3	97.8	99.1	99.7	97.2
**Cefepime**	**MIC**_**50**_	≤0.5	≤0.5	≤0.5	≤0.5	≤0.5	≤0.5	≤0.5	≤0.5	≤0.5	≤0.5
	**MIC**_**90**_	≤0.5	≤0.5	≤0.5	≤0.5	4	≤0.5	≤0.5	≤0.5	≤0.5	≤0.5
	**%S**	99.4	98.0	99.1	99.4	93.0	99.3	98.2	99.1	99.0	97.4
**Ceftriaxone**	**MIC**_**50**_	≤0.06	≤0.06	≤0.06	≤0.06	≤0.06	≤0.06	≤0.06	≤0.06	≤0.06	≤0.06
	**MIC**_**90**_	0.12	0.5	0.25	0.25	8	0.25	0.5	0.25	0.25	0.5
	**%S**	98.7	91.4	96.5	95.4	84.6	94.0	91.7	96.3	97.3	92.2
**Levofloxacin**	**MIC**_**50**_	0.06	0.06	0.06	0.06	0.06	0.06	0.06	0.06	0.06	0.06
	**MIC**_**90**_	0.25	0.5	0.5	0.5	≥16	1	1	1	0.5	1
	**%S**	99.4	93.4	96.3	95.5	86.2	90.7	92.9	94.7	97.3	92.7
**Minocycline**	**MIC**_**50**_	2	2	2	2	2	2	2	2	2	2
	**MIC**_**90**_	16	16	8	16	16	8	16	16	8	16
	**%S**	84.9	78.8	85.2	79.5	80.4	85.3	82.0	82.2	83.3	81.6
**Pip-taz**	**MIC**_**50**_	2	2	2	2	2	2	2	2	2	2
	**MIC**_**90**_	8	8	8	8	64	4	8	8	8	8
	**%S**	96.9	92.1	97.6	96.0	88.6	95.3	94.6	97.8	96.6	94.1
**Tigecycline**	**MIC**_**50**_	0.5	0.5	0.5	0.5	0.5	0.5	0.5	0.5	0.5	0.5
	**MIC**_**90**_	1	1	1	1	2	1	2	2	2	2
	**%S**	96.2	94.7	95.5	96.4	94.9	97.3	95.9	96.6	96.3	95.8
***Klebsiella oxytoca***		N = 57	N = 50	N = 176	N = 301	N = 279	N = 45	N = 145	N = 49	N = 68	N = 1170
**Amikacin**	**MIC**_**50**_	2	2	2	2	2	1	2	2	1	2
	**MIC**_**90**_	4	4	4	4	4	2	4	4	2	4
	**%S**	100	96.0	100	99.7	98.9	100	97.2	100	100	99.1
**Amoxi-clav**	**MIC**_**50**_	2	2	4	2	4	2	2	2	4	2
	**MIC**_**90**_	8	32	32	16	32	4	32	8	8	16
	**%S**	93.0	82.0	82.4	88.7	79.6	97.8	84.8	91.8	91.2	85.6
**Ampicillin**	**MIC**_**50**_	≥64	≥64	≥64	≥64	≥64	32	≥64	≥64	≥64	≥64
	**MIC**_**90**_	≥64	≥64	≥64	≥64	≥64	≥64	≥64	≥64	≥64	≥64
	**%S**	0.0	2.0	0.6	2.3	0.0	2.2	2.1	0.0	0.0	1.1
**Carbapenems**	**MIC**_**50**_	≤0.06	≤0.06	≤0.06	≤0.06	≤0.06	≤0.06	≤0.06	≤0.06	≤0.06	≤0.06
	**MIC**_**90**_	0.12	0.5	0.25	0.25	0.5	0.25	0.25	0.12	0.25	0.25
	**%S**	100	94.0	100	98.7	99.6	100	97.9	98.0	100	99.0
**Cefepime**	**MIC**_**50**_	≤0.5	≤0.5	≤0.5	≤0.5	≤0.5	≤0.5	≤0.5	≤0.5	≤0.5	≤0.5
	**MIC**_**90**_	≤0.5	2	2	1	2	≤0.5	1	≤0.5	≤0.5	1
	**%S**	100	96.0	98.9	98.7	98.6	100	96.6	100	100	98.5
**Ceftriaxone**	**MIC**_**50**_	≤0.06	≤0.06	≤0.06	≤0.06	≤0.06	≤0.06	≤0.06	≤0.06	≤0.06	≤0.06
	**MIC**_**90**_	0.25	4	4	2	16	0.25	4	0.5	0.5	4
	**%S**	93.0	86.0	83.0	88.7	77.8	97.8	85.5	91.8	92.6	85.6
**Levofloxacin**	**MIC**_**50**_	0.03	0.03	0.06	0.06	0.06	0.03	0.03	0.03	0.06	0.06
	**MIC**_**90**_	0.5	0.12	2	0.25	2	0.06	0.5	0.5	0.5	0.5
	**%S**	94.7	96.0	90.9	95.0	93.2	100	93.1	95.9	95.6	94.0
**Minocycline**	**MIC**_**50**_	1	1	1	2	2	1	1	1	1	1
	**MIC**_**90**_	2	16	4	8	8	2	4	4	8	8
	**%S**	98.2	86.0	90.3	89.0	87.1	95.6	91.0	95.9	85.3	89.7
**Pip-taz**	**MIC**_**50**_	2	1	1	1	1	1	1	1	2	1
	**MIC**_**90**_	4	32	≥256	8	≥256	2	8	2	8	16
	**%S**	94.7	88.0	86.9	92.4	86.0	97.8	91.7	98.0	94.1	90.4
**Tigecycline**	**MIC**_**50**_	0.25	0.25	0.25	0.25	0.5	0.25	0.25	0.25	0.5	0.25
	**MIC**_**90**_	0.5	1	1	1	2	0.5	1	0.5	2	1
	**%S**	100	98.0	99.4	99.0	98.2	100	97.9	100	100	98.9
***Serratia marcescens***		N = 87	N = 61	N = 302	N = 544	N = 609	N = 67	N = 475	N = 140	N = 136	N = 2421
**Amikacin**	**MIC**_**50**_	2	2	2	2	2	2	2	2	2	2
	**MIC**_**90**_	8	8	4	4	4	4	4	4	4	4
	**%S**	98.9	98.4	99.3	99.3	99.2	100	98.9	97.9	99.3	99.1
**Amoxi-clav**	**MIC**_**50**_	≥64	≥64	≥64	≥64	≥64	≥64	≥64	≥64	≥64	≥64
	**MIC**_**90**_	≥64	≥64	≥64	≥64	≥64	≥64	≥64	≥64	≥64	≥64
	**%S**	2.3	6.6	4.6	1.3	3.3	3.0	3.6	2.1	1.5	2.9
**Ampicillin**	**MIC**_**50**_	≥64	≥64	≥64	≥64	≥64	≥64	≥64	≥64	≥64	≥64
	**MIC**_**90**_	≥64	≥64	≥64	≥64	≥64	≥64	≥64	≥64	≥64	≥64
	**%S**	2.3	3.3	3.3	1.3	1.3	3.0	1.1	0.7	0.7	1.6
**Carbapenems**	**MIC**_**50**_	0.12	0.12	0.12	≤0.06	0.12	0.12	0.12	0.12	≤0.06	0.12
	**MIC**_**90**_	1	1	1	0.5	1	0.12	0.5	0.25	0.5	1
	**%S**	97.7	95.1	95.7	96.7	96.4	100	96.2	96.4	100	96.7
**Cefepime**	**MIC**_**50**_	≤0.5	≤0.5	≤0.5	≤0.5	≤0.5	≤0.5	≤0.5	≤0.5	≤0.5	≤0.5
	**MIC**_**90**_	≤0.5	1	1	4	4	≤0.5	1	≤0.5	1	2
	**%S**	100	100	98.0	95.4	97.2	100	97.7	97.9	100	97.4
**Ceftriaxone**	**MIC**_**50**_	0.25	0.25	0.25	0.25	0.25	0.25	0.25	0.25	0.25	0.25
	**MIC**_**90**_	2	2	4	32	16	1	4	4	2	8
	**%S**	88.5	86.9	88.7	78.3	77.8	92.5	83.6	86.4	89.7	82.6
**Levofloxacin**	**MIC**_**50**_	0.12	0.12	0.12	0.12	0.12	0.12	0.12	0.12	0.12	0.12
	**MIC**_**90**_	1	0.5	1	2	2	0.5	1	1	0.5	1
	**%S**	96.6	100	95.0	93.9	93.3	98.5	92.6	96.4	98.5	94.4
**Minocycline**	**MIC**_**50**_	2	4	4	4	4	2	4	4	4	4
	**MIC**_**90**_	8	8	8	8	8	8	8	8	8	8
	**%S**	86.2	77.0	75.8	74.1	81.0	88.1	78.9	89.3	73.5	78.7
**Pip-taz**	**MIC**_**50**_	2	1	1	2	1	1	1	1	2	1
	**MIC**_**90**_	8	8	4	8	8	4	4	4	4	8
	**%S**	98.9	96.7	97.0	93.6	94.1	98.5	96.2	97.1	97.8	95.5
**Tigecycline**	**MIC**_**50**_	1	1	1	1	1	1	1	1	1	1
	**MIC**_**90**_	2	2	2	2	2	2	2	1	2	2
	**%S**	98.9	91.8	97.4	96.5	95.4	97.0	95.4	97.9	91.9	95.9
***Enterobacter*spp.**		N = 227	N = 192	N = 770	N = 1351	N = 1500	N = 198	N = 1118	N = 339	N = 370	N = 6065
**Amikacin**	**MIC**_**50**_	2	2	2	2	2	2	2	2	2	2
	**MIC**_**90**_	4	4	2	4	4	2	4	2	4	4
	**%S**	100	99.5	99.5	98.7	98.1	99.5	99.2	98.8	98.6	98.9
**Amoxi-clav**	**MIC**_**50**_	≥64	≥64	≥64	≥64	≥64	≥64	≥64	≥64	≥64	≥64
	**MIC**_**90**_	≥64	≥64	≥64	≥64	≥64	≥64	≥64	≥64	≥64	≥64
	**%S**	4.8	4.7	7.3	3.3	3.7	2.5	5.5	6.2	5.1	4.7
**Ampicillin**	**MIC**_**50**_	≥64	≥64	≥64	≥64	≥64	≥64	≥64	≥64	≥64	≥64
	**MIC**_**90**_	≥64	≥64	≥64	≥64	≥64	≥64	≥64	≥64	≥64	≥64
	**%S**	2.2	6.3	5.8	2.7	2.9	3.5	2.8	2.9	3.0	3.3
**Carbapenems**	**MIC**_**50**_	≤0.06	≤0.06	≤0.06	≤0.06	0.12	≤0.06	≤0.06	≤0.06	≤0.06	≤0.06
	**MIC**_**90**_	0.5	1	0.5	0.5	1	0.5	0.5	0.5	0.5	0.5
	**%S**	100	97.4	98.6	97.9	96.1	99.5	97.9	97.3	97.8	97.6
**Cefepime**	**MIC**_**50**_	≤0.5	≤0.5	≤0.5	≤0.5	≤0.5	≤0.5	≤0.5	≤0.5	≤0.5	≤0.5
	**MIC**_**90**_	2	4	2	4	4	2	4	4	2	4
	**%S**	95.6	96.9	97.0	97.1	95.6	97.5	94.7	94.4	97.6	96.1
**Ceftriaxone**	**MIC**_**50**_	0.25	0.5	0.25	0.25	0.25	0.25	0.25	0.25	0.25	0.25
	**MIC**_**90**_	16	64	32	32	64	32	64	64	32	64
	**%S**	79.7	63.5	76.9	72.8	63.8	74.7	71.7	64.3	76.8	70.7
**Levofloxacin**	**MIC**_**50**_	0.03	0.06	0.06	0.06	0.06	0.03	0.06	0.06	0.06	0.06
	**MIC**_**90**_	0.25	0.5	0.5	1	4	0.5	4	≥16	0.5	2
	**%S**	97.8	93.2	93.9	93.0	89.4	98.0	86.8	85.0	93.5	91.0
**Minocycline**	**MIC**_**50**_	2	2	2	4	2	2	2	2	2	2
	**MIC**_**90**_	8	16	16	16	16	4	8	8	16	16
	**%S**	87.2	78.1	79.7	77.1	76.3	90.4	80.8	81.1	77.6	79.0
**Pip/taz**	**MIC**_**50**_	2	2	2	2	2	2	2	2	2	2
	**MIC**_**90**_	64	128	64	64	64	64	64	64	64	64
	**%S**	85.0	80.2	86.5	84.3	80.9	85.4	82.0	80.2	83.2	83.0
**Tigecycline**	**MIC**_**50**_	0.5	0.5	0.5	0.5	0.5	0.5	0.5	0.5	0.5	0.5
	**MIC**_**90**_	1	2	1	1	2	1	2	2	2	2
	**%S**	96.0	93.2	96.4	96.3	93.7	98.0	94.8	94.7	97.3	95.3

% S, % susceptible, *amoxi-clav* amoxicillin-clavulanate, *pip-taz* piperacillin-tazobactam, carbapenems = imipenem/meropenem.

**Table 4:** **Antimicrobial susceptibility for ESBL-positive *Escherichia coli *and *Klebsiella pneumoniae***

		**ESBL-producing*E. coli***	**ESBL-producing*K. pneumoniae***
		**N = 277**	**N = 584**
**Amikacin**	**MIC**_**50**_	4	16
	**MIC**_**90**_	16	32
	**%S**	94.9	77.1
**Amoxi-clav**	**MIC**_**50**_	16	16
	**MIC**_**90**_	≥64	≥64
	**%S**	29.6	29.1
**Ampicillin**	**MIC**_**50**_	≥64	≥64
	**MIC**_**90**_	≥64	≥64
	**%S**	0.7	0.0
**Carbapenems**	**MIC**_**50**_	≤0.06	0.12
	**MIC**_**90**_	0.25	16
	**%S**	98.2	74.0
**Cefepime**	**MIC**_**50**_	32	16
	**MIC**_**90**_	≥64	≥64
	**%S**	34.3	48.1
**Ceftriaxone**	**MIC**_**50**_	≥128	64
	**MIC**_**90**_	≥128	≥128
	**%S**	2.2	3.1
**Levofloxacin**	**MIC**_**50**_	≥16	≥16
	**MIC**_**90**_	≥16	≥16
	**%S**	5.8	19.9
**Minocycline**	**MIC**_**50**_	4	4
	**MIC**_**90**_	≥32	≥32
	**%S**	61.0	54.8
**Pip-taz**	**MIC**_**50**_	4	128
	**MIC**_**90**_	128	≥256
	**%S**	79.4	40.1
**Tigecycline**	**MIC**_**50**_	0.25	1
	**MIC**_**90**_	0.5	2
	**%S**	100	92.1

% S, % susceptible, *amoxi-clav* amoxicillin-clavulanate, *pip-taz* piperacillin-tazobactam, carbapenems = imipenem/meropenem.

### *Klebsiella pneumoniae* and *K. oxytoca*

Non-ESBL producing *K. pneumoniae* and *K. oxytoca* (Table 3) were highly susceptible (>90%) to cefepime, carbapenems, amikacin, and tigecycline regardless of region; susceptibility was only slightly lower for ceftriaxone, levofloxacin and piperacillin-tazobactam. There was a marked increase in the MIC_90_ for several antimicrobials against *Klebsiella* spp. collected from the Middle Atlantic region. In addition, there was a higher frequency (11%) of *K. pneumonia* isolates that produced ESBL (n = 584) compared with the numbers of ESBL-producing *E. coli* isolates, and the highest numbers of ESBL-producing *K. pneumonia* isolates were recovered from the Middle Atlantic region (17.7%, 268/1518). The ESBL-producing isolates were mostly susceptible to amikacin (77.1%) and carbapenems (74.0%) and were highly susceptible (92.1%) to tigecycline (Table 4).

### *Serratia marcescens*

*S. marcescens* isolates were highly susceptible to cefepime (97.4%), carbapenems (96.7%), tigecycline (95.9%), and levofloxacin (94.4%) (Table 3). Amikacin (MIC_50_ 2 mg/L, MIC_90_ 8 mg/L, 99.1% susceptible) and piperacillin-tazobactam (MIC_50_ 1 mg/L, MIC_90_ 8 mg/L, 95.5% susceptible) were also active, but susceptibilities to ceftriaxone and minocycline varied among the census regions. For example, there were considerably higher numbers of ceftriaxone-resistant isolates recovered from the East North Central and Middle Atlantic region when compared with recovery in New England (78.3% and 77.8% versus 92.5% susceptible). In addition, resistance to minocycline was most prevalent in the West South Central region (73.5% susceptible).

### *Enterobacter* spp.

*Enterobacter* spp. were highly susceptible to amikacin (98.9%), carbapenems (97.6%), and cefepime (96.1%), and tigecycline (MIC_50_ 0.5 mg/L, MIC_90_ ≤ 2 mg/L, 95.3%) was also highly effective (Table 3). In contrast, susceptibility to ceftriaxone, levofloxacin, and minocycline was more variable, and the differences were most pronounced when comparing MIC_90_ values. For example, isolates from the Pacific region were highly susceptible (79.7%) to ceftriaxone, while organisms from the Mountain region were considerably more resistant (63.5% susceptibility).

## Discussion

Antimicrobial resistance among Gram-negative organisms continues to be a major concern, especially considering the potential for the rapid spread of resistance mechanisms and the limited treatment options that result. In this study, we examined the activity of β-lactam, aminoglycoside, and fluoroquinolone antimicrobials against Enterobacteriaceae and *A. baumannii* isolates collected from nine regions within the USA. We also examined the susceptibility of each isolate to tigecycline, a glycylcycline licensed to treat infections caused by a broad spectrum of microorganisms, many of which have acquired resistance to treatment with traditional antimicrobials. In addition, Halstead et al. [2007] published a comprehensive report of antimicrobial susceptibilities of Gram-negative isolates collected from the USA during 2004 and 2005 [8] and we extend the study by determining the antimicrobial susceptibilities of a more diverse group of isolates that highlights ongoing nationwide changes in resistance patterns. It should be noted, however, that we failed to test each appropriate Gram-negative isolate for susceptibility to imepenem and meropenem, which forced us to incorporate our findings into the broader category of carbapenem resistance. However, we are confident this shortcoming did not prevent valid comparison of our results with previous findings.

Several other recent studies also determined the susceptibilities of Gram-negative organisms to multiple antimicrobial agents [2,12,13], with results similar to this study. For example, we detected similarly high prevalence of sensitivity of *K. oxytoca* and non-ESBL producing *E. coli* to levofloxacin, piperacillin-tazobactam, and ceftriaxone. In addition, *Enterobacter* spp., *K. oxytoca*, and *S. marcescens* were highly susceptible to the carbapenems, while the non-ESBL-producing *E. coli* and *K. pneumoniae* isolates were almost universally susceptible to the carbapenems. However, small numbers of carbapenem-resistant organisms were recovered from each genus, which also supports previous findings that highlight the necessity for continued monitoring for carbapenem resistance. In addition, *A. baumannii* isolates that were highly resistant to multiple other antimicrobial agents were also highly resistant to the carbapenems (imipenem/meropenem), a result which has been previously reported [14]. This is especially disconcerting since the only option for effective treatment of these highly resistant organisms, especially MDR *A. baumannii* infections, may be dependent on salvage agents such as colistin which introduce a host of additional complications [14,15].

Comparing the susceptibility patterns to previous findings [8] also revealed several important trends. Most notably, the prevalence of resistant organisms remained essentially unchanged in the East South Central, Middle Atlantic, and Pacific regions; the prevalence of organisms that were resistant to levofloxacin also decreased significantly (p < 0.01). Significant (p < 0.01) increases in susceptibility were identified in 8 region/organism/antimicrobial agent combinations between 2004–2005 and 2005–2011, 4 of these occurring in the Middle Atlantic region. Significant decreases in susceptibility were noted in 26 cases over the same time interval; 12 of these occurred in East North Central while 8 were noted in South Atlantic. Notably, in South Atlantic, *K. pneumoniae* susceptibility to levofloxacin, amikacin, amoxicillin-clavulanate, cefepime, minocycline and piperacillin-tazobactam decreased significantly. In East North Central, *A. baumannii* susceptibility to amikacin, ceftriaxone, levofloxacin, minocycline and piperacillin-tazobactam reduced significantly while *E. coli* susceptibility to amoxicillin-clavulanate, cefepime, levofloxacin and minocycline decreased significantly. These findings highlight the importance of local efforts to monitor changing antimicrobial susceptibility patterns for accurately guiding appropriate treatment regimens. In addition, evaluating the infection control practices in regions where the prevalence of antibiotic resistant organisms has not increased significantly may provide important insight into effective methods for curbing emerging resistance in other regions.

Finally, despite the lack of established efficacy standards for predicting the success of treatment with tigecycline, our findings confirmed and extended previous observations of high *in vitro* activity against Enterobacteriaceae (*E. coli*, 100% susceptible; *Enterobacter*, 98.4% susceptible; ESBL-positive *K. pneumoniae*, 97.9%) and also *A. baumannii* (94.4% susceptible at ≤ 2 mg/L) [16]. Therefore, additional studies to determine the efficacy of tigecycline *in vivo*, especially for treating infections with MDR organisms, are warranted.

## Competing interests

GAD: is a paid investigator for the Tigecycline Evaluation and Surveillance Trial.

SMC: Gundersen Health System received modest monetary compensation from Pfizer Inc. for providing MIC values of isolates collected in-house. However, SMC received no direct financial support and also has no industrial/commercial relationships that may be interpreted to pose a conflict of interest.

MJD: is an employee of Pfizer Inc.

## Author’s contributions

GAD was involved in the collection of data for this study, the analysis and interpretation of these data, and in drafting and revising the content of this manuscript. GAD has given approval for this version of the manuscript to be published. SMC was involved in the collection of data for this study, the analysis and interpretation of these data, and in drafting and revising the content of this manuscript. SMC has given approval for this version of the manuscript to be published. MD was involved in the concept, design and execution of the T.E.S.T. study. MD has also been involved in the revision of this manuscript, and given approval for this version of the manuscript to be published. All authors read and approved the final manuscript.
